# Reversible Addition-Fragmentation Chain Transfer Polymerization of Acrylonitrile under Irradiation of Blue LED Light

**DOI:** 10.3390/polym9010004

**Published:** 2016-12-26

**Authors:** Zhicheng Huang, Lifen Zhang, Zhenping Cheng, Xiulin Zhu

**Affiliations:** Suzhou Key Laboratory of Macromolecular Design and Precision Synthesis, Jiangsu Key Laboratory of Advanced Functional Polymer Design and Application, State and Local Joint Engineering Laboratory for Novel Functional Polymeric Materials, Department of Polymer Science and Engineering, College of Chemistry, Chemical Engineering and Materials Science, Soochow University, Suzhou 215123, China; huangzc_1991@163.com (Z.H.); xlzhu@suda.edu.cn (X.Z.)

**Keywords:** reversible addition-fragmentation transfer (RAFT), blue LED, polyacrylnitrile, photocatalysis

## Abstract

Compared to unhealthy UV or γ-ray and high-energy-consumption thermal external stimuli, the promising light emitting diode (LED) external stimulus has some outstanding technological merits such as narrow wavelength distribution, low heat generation and energy consumption, and safety for human beings. In this work, a novel reversible addition-fragmentation transfer (RAFT) polymerization system for acrylonitrile (AN) was developed under the irradiation of blue LED light at room temperature, using 1,2,3,5-tetrakis(carbazol-9-yl)-4,6-dicyanobenzene (4CzIPN) as a novel radical initiator and 2-cyanoprop-2-yl-1-dithionaphthalate (CPDN) as the typical chain transfer agent. Well-defined polyacrylonitrile (PAN) with a controlled molecular weight and narrow molecular weight distribution was successfully synthesized. This strategy may provide another effective method for scientific researchers or the industrial community to synthesize a PAN-based precursor of carbon fibers.

## 1. Introduction

It is generally known that carbon fibers [1,2,3,4] are an important material for the development of advanced technology. Due to the outstanding properties of carbon fibers, among which are a low density, high axial strength, high modules, excellent fatigue resistance, high temperature resistance and corrosion resistance, they have been utilized in varieties of military and civilian industries, including aerospace, aviation, machineries, textiles, sports equipment and leisure products. Compared with carbon fibers based on other precursors, polyacrylonitrile (PAN)-based carbon fibers [5,6,7,8] with impressive tensile strength and mechanical properties have occupied the greatest market share in the carbon fiber field. However, the experimental measured values of the tensile strength of PAN-based carbon fibers are far below the theoretically predicted values due to the fact that conventional PAN precursors polymerized by solution, suspension or emulsion polymerization have impurities and defects [1]. Therefore, a high-quality PAN precursor with a low molecular weight distribution is essential to prepare PAN-based carbon fibers.

Reversible deactivation radical polymerization (RDRP) [9,10,11,12,13,14,15,16,17,18,19,20,21,22,23,24] techniques have presented versatility in macromolecular precision synthesis, topologies design, and control over molecular weight and molecular weight distribution in the past few decades. Although atom transfer radical polymerization (ATRP) also shows robust skills in the control of polymerization of acrylonitrile (AN) [25,26,27,28], the transition metal−based catalyst residue would restrict applications in the field of PAN-precursor carbon fibers. On the contrary, reversible addition-fragmentation chain transfer (RAFT) polymerization, as one of most efficient RDRP techniques, would successfully circumvent this thorny issue. Because of the high reactivity of AN and the poor solubility of PAN, there are some hurdles in achieving good control of the polymerization of AN [29]. Nevertheless, several efforts also have been reported to synthesize a high-molecular-weight PAN precursor with a low molecular weight distribution with the assistance of the RAFT technique. As early as 2003, Tang et al. [30] reported that PAN and block copolymers were prepared via the RAFT polymerization technique using 2-cyanoethyl dithiobenzoate (CED) as the transfer agent at 60 °C. A bifunctional RAFT agent, 1,4-(2-(carbazole-9-carbodithioate)-2-methl-propionic acid)phenyl ester (BCCDP) was employed to mediate RAFT polymerization of AN at 75 °C by our group [31]. Additionally, well-defined PAN with a high-viscosity-average molecular weight and a low molecular weight distribution was synthesized. Buchmeiser’s group [32] indicated that controlled RAFT homopolymerization of AN was accomplished with the use of 2-cyano-2-propyl dodecyltrithiocarbonate (CPDT) at 90 °C. Very recently, Matyjaszewski et al. [33] demonstrated that controlled RAFT polymerization of AN occurred in an aqueous solution of sodium thiocyanate with 4-cyano-4-(phenylcarbonothioylthio)pentanoic acid (CPAD) as the chain transfer agent at 65 °C. Lu et al. [34] reported that the synthesis of isotactic PAN by γ-ray irradiation induced eth polymerization of AN. As reviewed above, the external stimulus for the polymerization of AN is either thermally induced with high energy consumption or induced by unhealthy γ-ray irradiation.

Unfortunately, both thermally stimulated and unhealthy γ-ray−irradiated [34,35,36]. polymerization systems have contravened the demand for more sustainable “green” and economical chemical processes in this resource-exhausting society. Moreover, some side reactions could not be avoided in these polymerization processes due to the high-energy-input external stimulus. On the other hand, the visible light emitting diode (LED) photocatalysis strategy is unique in meeting the energetic requirements to initiate polymerization compared to thermal or γ-ray counterparts. Additionally, the visible LED photocatalysis strategy [37,38,39,40,41,42,43] has been considered as an ideal external stimulus for RDRP due to the typical impressive advantages of LED light, including low wavelength distribution, low heat generation and energy consumption, eco-friendliness and lack of pollution, easy operation and safety for human beings. In this work, by combining the advantages of RAFT polymerization and the visible LED photocatalysis strategy, we developed a RAFT polymerization of an AN system under visible blue LED irradiation at room temperature, using 1,2,3,5-tetrakis(carbazol-9-yl)-4,6-dicyanobenzene (4CzIPN) [44] as a novel radical initiator and 2-cyanoprop-2-yl-1-dithionaphthalate (CPDN) as the typical chain transfer agent. PAN with a low molecular weight distribution was successfully prepared under blue LED stimulation at room temperature. Furthermore, the polymerization kinetics further confirmed the “living”/controlled features of this RAFT polymerization system. In addition, the high chain-end functional fidelity has been verified by 1H nuclear magnetic resonance (NMR) and matrix-assisted laser desorption/ionization time-of-flight mass spectrometry (MALDI-TOF MS).

## 2. Experimental Section

### 2.1. Materials

Acrylonitrile (AN, 99+%, Shanghai Chemical Reagents Co. Ltd., Shanghai, China) was purified by passing through a neutral alumina column. *N*,*N*-dimethylformamide (DMF, analytical reagent, Shanghai Chemical Reagents Co. Ltd., Shanghai, China) and dimethylsulfoxide (DMSO, analytical reagent, Shanghai Chemical Reagents Co. Ltd., Shanghai, China) were dried using 4 Å molecular sieve. CPDN [45,46] and 4CzIPN [47,48] were synthesized according to the literatures. 2,2′-Azobis(2-methylpropionitrile) (AIBN) (Chemically pure, Shanghai Chemical Reagents Co. Ltd., Shanghai, China) was recrystallized from ethanol, and dried in a vacuum oven. Diphenyl(2,4,6-trimethylbenzoyl)phosphine oxide (TPO) was obtained from J&K China chemical Ltd. (Beijing, China) and used as received. All other chemicals were purchased from Shanghai Chemical Reagents Co. Ltd. and were used as received unless mentioned.

### 2.2. Characterizations

The number-average molecular weight (*M*_n,GPC_) and molecular weight distribution (*M*_w_/*M*_n_) values of the obtained polymers were determined by a TOSOH HLC-8320 gel permeation chromatograph (GPC) equipped with a refractive-index detector (Tosoh Bioscience Shanghai Co. Ltd., Shanghai, China), using TSK gel guardcolumn superMP-N (4.6 mm × 20 mm) and two TSK gel supermultipore HZ-N (4.6 mm × 150 nm) with measurable molecular weights ranging from 10^2^ to 10^6^ g·mol^−1^. DMF with 10 mmol·L^−1^ of LiBr was used as eluent at a flow rate of 1.0 mL·min^−1^ at 40 °C. GPC samples were injected using a TOSOH plus autosampler and calibrated with PS standards purchased from TOSOH (Tosoh Bioscience Shanghai Co. Ltd., Shanghai, China). The ^1^H Nuclear Magnetic Resonance (NMR) spectra of polymers was recorded on a Bruker 300 MHz nuclear magnetic resonance instrument (Bruker Daltonics Inc., Billerica, MA, USA) using DMSO-d_6_ as the solvent and tetramethylsilane (TMS) as the internal standard. Matrix assisted laser desorption/ionization time-of-flight mass spectra were recorded with an UltrafleXtreme MALDI-TOF mass spectrometer (Bruker Daltonics Inc., Billerica, MA, USA) equipped with a 1000 Hz smart beam-II laser.

### 2.3. Typical Procedures for Photoinduced RAFT Polymerization of AN

A typical polymerization procedure with the molar ratio of [AN]_0_:[CPDN]_0_:[4CzIPN]_0_ = 400:1:0.01 was carried out in a pre-dried Schlenk tube or a clean ampoule. The reaction mixture was prepared by adding AN (1.0 mL, 15.2 mmol), CPDN (10.29 mg, 3.8 × 10^−2^ mmol), 4CzIPN (0.30 mg, 3.8 × 10^−4^ mmol), and solvent DMSO (1.0 mL). The polymerization system was deoxygenized by freeze-pump-thaw cycle for three times, and sealed up subsequently. The polymerization was conducted under blue LED irradiation (λ_max_ = 458 nm, 0.85 mW·cm^−2^) at room temperature. After the predetermined time, 0.5 mL of reaction solution was removed via syringe (in Schlenk tube) or pipette (in ampoule). Then 0.1 mL of the reaction solution was analyzed by ^1^H NMR for monomer conversion and another 0.1 mL was dissolved in DMF for analysis by GPC for number-average molecular weight (*M*_n,GPC_) and molecular weight distribution (*M*_w_/*M*_n_) values.

## 3. Results and Discussion

Based on our previous work, 4CzIPN, as a novel organic photocatalyst in metal-free ATRP, can also solely initiate polymerization under blue LED irradiation [44]. Therefore, we employed 4CzIPN as a photoinitiator in the RAFT polymerization of AN mediated by CPDN in this work for the first time. Well-defined PANs with controlled molecular weights and molecular weight distributions were synthesized by a series of polymerizations under varied conditions.

The results are shown in Table 1. In the absence of 4CzIPN (entry 1 in Table 1) or blue LED irradiation (entry 3 in Table 1), no polymers were obtained even after 24 h of polymerization time, while 55.1% of the monomer conversion was achieved in 3.5 h to yield PAN with a high-number-average molecular weight and a broad molecular weight distribution (*M*_n,GPC_ = 155,700 g·mol^−1^, *M*_w_/*M*_n_ = 2.26) under blue LED irradiation (entry 2 in Table 1) in the absence of CPDN. It indicates that 4CzIPN can indeed serve as a novel photoinitiator and CPDN as a chain transfer agent, making them great choices for well-controlled RAFT polymerization of AN. The polymerizations in the presence of 4CzIPN resulted in PANs with calibrated number-average molecular weights [28] close to the corresponding theoretical ones and narrow molecular weight distributions. Although the level of molecular weight control with 4CzIPN was similar to that of unhealthy UV-sensitive TPO (entry 5 in Table 1) and thermally unstable AIBN (entry 6 in Table 1), visible-light-sensitive 4CzIPN (entry 4 in Table 1) as a novel initiator is more suitable for CPDN-mediated RAFT polymerization of AN, due to the lower energy consumption and being environmentally friendly. Additionally, the effect of the degree of polymerization targets for blue-LED−photoinduced RAFT polymerization of AN was further investigated (entries 7−10 in Table 1). Moreover, with the increase of the molecular weights of the PANs, the trend of higher molecular weight distribution was also observed. It is well known that the establishment of reversible dynamic equilibrium between active species with dormant species in RAFT polymerization would be retarded due to the diffusion control [49] of free radicals in a high-viscosity mixture of high-molecular-weight PANs.

**Figure 1:**
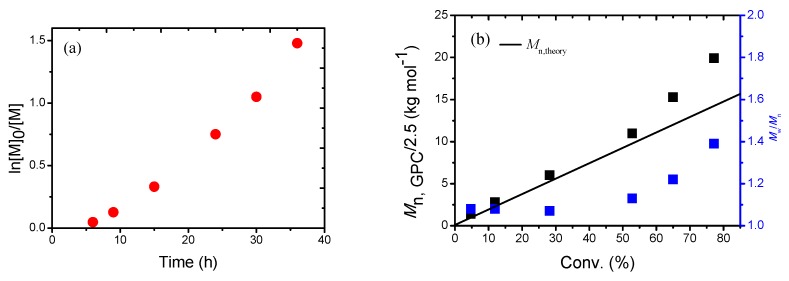
ln([*M*]_0_/[*M*]) as a function of time (**a**), number-average molecular weight (*M*_n,GPC_) and molecular weight distribution (*M*_w_/*M*_n_) versus monomer conversion (**b**) for photoinduced RAFT polymerization of AN under blue LED irradiation. Polymerization conditions: [AN]_0_:[CPDN]_0_:[4CzIPN]_0_ = 400:1:0.01, *V*_AN_ = 5.0 mL, *V*_DMSO_ = 10.0 mL, under blue LED irradiation (λ_max_ = 458 nm, 0.85 mW·cm^−2^).

To deeply understand the polymerization behaviors of AN, the polymerization kinetics with a molar ratio of [AN]_0_:[CPDN]_0_:[4CzIPN]_0_ = 400:1:0.01 were carried out under blue LED irradiation at room temperature. An obvious induction period was observed (Figure 1a), which may be attributed to the slow decomposition of intermediate radical species. The pseudo-first-order polymerization kinetic plot indicated that the radical concentration remained approximately constant during the RAFT polymerization period. The calibrated number-average molecular weights which approached the corresponding theoretical ones almost linearly increased with monomer conversion (Figure 1b). In addition, the obtained PANs all have moderately narrow molecular weight distributions (*M*_w_/*M*_n_ < 1.4). All evidence above confirmed the “living”/controlled features of this novel blue-LED−photoinduced RAFT polymerization of AN.

**Figure 2:**
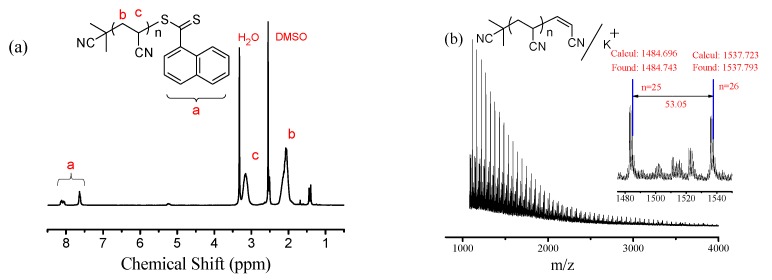
^1^H NMR spectrum (**a**) of PAN (*M*_n,GPC_ = 5200 g·mol^−1^, *M*_w_/*M*_n_ = 1.09) and MALDI-TOF MS (**b**) of PAN (*M*_n,GPC_ = 7000 g·mol^−1^, *M*_w_/*M*_n_ = 1.08) synthesized by photoinduced RAFT polymerization under blue LED irradiation.

For further insight into the “living” feature of the blue-LED−photoinduced RAFT polymerization of AN, the chain-end group of PAN was analyzed by ^1^H NMR in DMSO-d_6_ as shown in Figure 2a. The signals at chemical shifts δ = 7.6–8.2 ppm (a in Figure 2a) were assigned to the protons of naphthyl from CPDN. The signals at chemical shifts δ = 2.1 ppm (b in Figure 2a) and δ = 3.1 ppm (c in Figure 2a) were assigned to the protons of methylene and methine in the PAN backbone, respectively. In addition, well-defined PAN prepared by blue-LED−photoinduced RAFT polymerization was analyzed by MALDI-TOF MS (Figure 2b). The main series of peaks with the same interval (53.05 of *m*/*z*, representing an AN repeat unit) were assigned to PAN with an isobutyronitrile group at the α chain end and vinyl at the ω chain end. Loss of the CPDN fragment at the ω chain end of PAN was attributed due to a weak carbon-sulfur bond cleavage and removal from the PAN chain end during MALDI-TOF MS testing, as previously report [50]. Therefore, it clearly demonstrated that the blue-LED−photoinduced RAFT polymerization of AN was successfully mediated by CPDN and initiated by 4CzIPN. In addition, according to the results discussed above, the possible polymerization mechanism of this system was proposed. In this system, 4CzIPN acted as a free radical “initiator”. Namely, the initial free radicals should be generated from 4CzIPN under blue LED irradiation to initiate the typical RAFT polymerization of AN in the presence of CPDN, although the precise structure of the free radicals is unclear so far.

## 4. Conclusions

The organic photocatalyst 4CzIPN was successfully used in the RAFT polymerization of AN under blue LED irradiation at room temperature with CPDN as the chain transfer agent. It is worth mentioning that this strategy may provide an approach for scientific researchers and the industrial community to easily synthesize well-defined PAN-based precursors for carbon fibers.

## Figures and Tables

**Table 1:** Results of photoinduced RAFT polymerization of AN under blue LED irradiation ^a^.

Entry	R	Time (h)	Conv. ^b^ (%)	*M*_n,GPC_^c^ (g/mol)	*M*_n,GPC_/2.5 ^d^ (g/mol)	*M*_n,th_^e^ (g/mol)	*M*_w_/*M*_n_^c^
1	[AN]_0_/[CPDN]_0_ = 400/1	24	N.A.				
2	[AN]_0_/[4CzIPN]_0_ = 400/0.01	3.5	55.1	155,700	-	-	2.26
3 ^f^	[AN]_0_/[CPDN]_0_/[4CzIPN]_0_ = 400/1/0.01	24	N.A.				
4	[AN]_0_/[CPDN]_0_/[4CzIPN]_0_ = 400/1/0.01	24	54.7	25,200	10,100	11,600	1.12
5 ^g^	[AN]_0_/[CPDN]_0_/[TPO]_0_ = 400/1/0.01	24	25.7	14,000	5600	5400	1.07
6 ^h^	[AN]_0_/[CPDN]_0_/[AIBN]_0_ = 400/1/0.01	24	70.3	37,400	15,000	15,200	1.11
7	[AN]_0_/[CPDN]_0_/[4CzIPN]_0_ = 400/1/0.01	22	65.5	45,200	18,100	21,100	1.29
8	[AN]_0_/[CPDN]_0_/[4CzIPN]_0_ = 800/1/0.02	9	44.4	32,400	13,000	19,100	1.20
9	[AN]_0_/[CPDN]_0_/[4CzIPN]_0_ = 1,000/1/0.025	9	47.9	45,500	18,200	25,700	1.32
10	[AN]_0_/[CPDN]_0_/[4CzIPN]_0_ = 1,500/1/0.075	4	58.7	75,200	30,100	46,900	1.46

Polymerization conditions: ^a^ *V*_AN_ = 1.0 mL, *V*_DMSO_ = 1.0 mL, under blue LED irradiation (λ_max_ = 458 nm, 0.85 mW·cm^−2^) at room temperature; ^b^ Determined by ^1^H NMR; ^c^ Determined by GPC; ^d^ Calibrated number-average molecular weights were proposed by Matyjaszewski et al. [28]; ^e^ *M*_n,th_ = [AN]_0_/[CPDN]_0_ × Conv. × *M*_AN_ + *M*_CPDN_; ^f^ Polymerization run in dark; ^g^ Polymerization under UV irradiation (λ_max_ = 365 nm, 0.90 mW·cm^−2^); ^h^ Polymerization at 70 °C.
